# Serum IL-17F as a biomarker of infection-independent cirrhosis progression

**DOI:** 10.3389/fimmu.2025.1671288

**Published:** 2025-10-22

**Authors:** Georgios Konstantis, Moritz Passenberg, Clara Guntlisbergen, Andreas Schütte, Björn Jung, Nargiz Nuruzade, Florian Seltsam, Dieter P. Hoyer, Jan Best, Katharina Willuweit, Hartmut H. Schmidt, Sabrina Guckenbiehl, Jassin Rashidi-Alavijeh

**Affiliations:** ^1^ Department of Gastroenterology, Hepatology and Transplant Medicine, Medical Faculty, University of Duisburg-Essen, Duisburg, Germany; ^2^ Department of General, Visceral and Transplantation Surgery, University Hospital Essen, Essen, Germany

**Keywords:** ACLF (acute on chronic liver failure), liver fibrosis and cirrhosis, IL17, IL23, IL17F

## Abstract

**Background:**

Cirrhosis-associated immune dysfunction (CAID) is characterized by a dysregulated immune response involving both systemic inflammation and immunosuppression, frequently culminating in acute-on-chronic liver failure (ACLF). The IL-23/IL-17 signaling axis is a key inflammatory pathway implicated in various immune-mediated diseases, yet its specific role in cirrhosis remains poorly understood.

**Methods:**

In this prospective observational study, we analyzed circulating levels of IL-23, IL-17A, IL-17E, IL-17F, IL-1β, and IL-1RA in 127 patients with compensated cirrhosis, acute decompensation (AD), or ACLF in the absence of active infection. Cytokines were quantified by ELISA and correlated with disease severity, organ dysfunction, and clinical outcomes. Multivariable ordinal and multinomial regression analyses were performed to assess associations between cytokines and cirrhosis progression.

**Results:**

Levels of IL-17F, IL-23, and IL-1β levels were significantly lower in patients with AD and ACLF compared to those with compensated cirrhosis. In contrast, IL-17A, IL-17E, and IL-1RA levels remained largely unchanged. IL-17F was independently associated with reduced odds of progression to AD or ACLF, suggesting a possible protective role. IL-17F also retained an inverse association with disease severity in adjusted ordinal regression models.

**Conclusion:**

IL-17F expression declines progressively with increasing severity of liver disease, independently of IL-17A. These findings support a distinct, possibly immunoregulatory role for IL-17F in cirrhosis progression, and warrant further mechanistic investigation into its functional relevance as a biomarker for disease stratification or as a therapeutic target.

## Introduction

Cirrhosis-associated immune dysfunction (CAID) is a severe complication of cirrhosis, characterized by both immunodeficiency and systemic inflammation resulting from persistent and inappropriate activation of immune cells (1). With disease progression, systemic inflammation shifts toward immunosuppression, driven by mechanisms such as impaired hepatic immune surveillance and reduced immune cell function (2–5). The prevailing hypothesis suggests that bacterial translocation from the gut leads to increased immune activation, promoting elevated levels of circulating pro-inflammatory cytokines and upregulation of activation markers on immune cells (1, 6).

The severe and often rapid progression to acute-on-chronic liver failure (ACLF) is closely associated with this sterile inflammation and immune dysregulation (2, 4, 7–9). This highlights the urgent need for a better understanding of the underlying immunological milieu, as no effective medical therapy is currently available, and liver transplantation remains the only curative option (10–12). Within this framework, numerous studies have investigated cytokine profiles and immune cell dynamics during episodes of acute decompensation. However, accurately characterizing baseline systemic inflammation remains difficult, largely due to the presence of confounding variables, most notably infectious precipitants. This distinction is crucial, as infection-induced inflammation may mask the underlying immune phenotype of cirrhosis. Consequently, the molecular mechanisms underpinning sterile inflammation remain unclear (13, 14).

In this context, the IL-23/IL-17 signaling axis represents a potentially important pathway (15). This cytokine network is well established in the pathogenesis of immune-mediated diseases such as psoriasis, inflammatory bowel disease and rheumatoid arthritis (16–19). However, in the context of chronic liver disease, the IL-23/IL-17 axis has received limited attention. A study from 2013 demonstrated that elevated serum levels of IL-23 and IL-17 in patients with primary biliary cholangitis were associated with more advanced clinical stages and elevated gamma-glutamyl transferase (GGT) levels (20).Another study found significantly higher plasma IL-23 concentrations in patients with metabolic dysfunction-associated steatohepatitis (MASH), while increased IL-17A and decreased IL-10 levels were observed in patients with chronic hepatitis C compared to healthy controls (21). Despite thesexfindings, the mechanistic role of this cytokine axis in the immunopathogenesis of acute decompensation remains poorly defined.

In the present study, we aimed to investigate the clinical relevance of key circulating cytokines within the IL-23/IL-17 signaling pathway in patients with acutely decompensated cirrhosis, specifically in the absence of infectious triggers. We also measured IL-17E (IL-25) and IL-1 receptor antagonist (IL-1RA) to capture regulatory nodes within this pathway. IL-17E promotes type-2 immunity, counter-regulates Th17 activity, and has been shown to inhibit Th17-mediated immunity, while IL-1RA is the endogenous antagonist of IL-1 receptor signaling (22–25). We examined the potential role of this inflammatory axis in promoting the transition from stable cirrhosis to ACLF. Additionally, we sought to determine whether these cytokines are associated with complications such as ascites and hepatic encephalopathy, and whether they correlate with laboratory markers of hepatic synthetic function.

## Study population

Between November 2022 and April 2023, consecutive patients admitted to the University Hospital Essen, Germany, with acute decompensation of cirrhosis and/or ACLF according to the criteria of the Chronic Liver Failure–European Association for the Study of the Liver (CLIF-EASL) consortium have been prospectively included in our cirrhosis cohort study (3). In 2024, the cohort was expanded to include patients with compensated or stable decompensated cirrhosis.

Cirrhosis was defined as the presence of at least two of the following: i) histological confirmation on liver biopsy, ii) laboratory abnormalities consistent with cirrhosis, or iii) radiological signs indicative of cirrhosis and portal hypertension. Acute decompensation of cirrhosis was identified by the occurrence or worsening of specific complications, including hepatic encephalopathy [graded according to the West-Haven criteria (26)], gastrointestinal bleeding, bacterial infections, or moderate to severe ascites [grade 2–3, classified according to recommendations of the International Club of Ascites (27)]. The diagnosis of ACLF was established based on the criteria defined by the CLIF-EASL consortium (11).

Patients who met the following exclusion criteria were not included: acute liver failure, known congenital coagulation disorders, pregnancy, HIV seropositivity, or evidence of disseminated malignancy, infection diagnosed 48 hours after admission. AD of chronic liver disease and ACLF were classified and graded based on the criteria established in the CANONIC study (28). Baseline comorbidities and medication lists were screened for chronic immune-mediated inflammatory diseases with known IL-17 activation (e.g., psoriasis, rheumatoid arthritis) and for IL-17/IL-23–targeted biologics. Patients meeting any of these criteria were excluded from the analysis.

Clinical data and biomaterials were obtained from patients with AD or ACLF at baseline and at the 12-week follow-up. For patients with compensated or stable decompensated cirrhosis, data and biomaterials were collected also at baseline and subsequently at least every three months during follow-up or at the onset of acute decompensation or ACLF for up to 12 months. This study was conducted according to the guidelines of the Declaration of Helsinki and was approved by the ethics committee of the Medical Faculty of the University of Duisburg-Essen (*23-11088-BO and 22-10813-BO*).

### Quantification of cytokines

Serum samples were collected at predefined baseline and follow-up time points. Concentrations of IL-23, IL-1β, IL-1RA, IL-17F, IL-17E, and IL-17A were quantified using the Human IL-22 Quantikine ELISA Kit (R&D Systems, Minneapolis, MN), following the manufacturer’s instructions. Optical density was measured at 450 nm using an EnVision plate reader. Values falling outside the detection range were assigned the maximum or minimum values from the respective standard curves. For statistical analysis, concentrations below the detection limit were imputed as 0.5 x the lowest detectable value of the corresponding standard curve.

### Statistical analyses

Statistical analyses were conducted using R Studio (Version 4.2). Group differences were assessed using χ² (chi-squared) tests for categorical variables, or Fisher’s exact test when expected cell counts were below five. For continuous variables, the Kruskal–Wallis test was applied in cases of non-normal distribution. Normality was evaluated using appropriate tests, and non-parametric methods were chosen where necessary. Due to the small number of events, mortality was analyzed using binary logistic regression. A p-value of less than 0.05 was considered statistically significant. Associations between outcomes and continuous or categorical variables were examined using linear and logistic regression models, respectively. Significant associations identified in univariate analyses were further analyzed through multivariate regression models. These multivariate models were adjusted for predefined variables that, based on clinical expertise and relevant literature, were considered potential confounders influencing the univariate results. Multinomial logistic regression was applied to examine associations between cytokines and cirrhosis stage (compensated, AD, ACLF), without assuming an inherent order. In addition, ordinal regression was used to model the progression from compensated cirrhosis to AD and ACLF, reflecting the sequential nature of disease worsening. Model assumptions were tested for validity. Both regression models were adjusted for potential confounders identified in univariable analyses. Cytokine concentrations were log-transformed to approximate a normal distribution and reduce the influence of outliers.

## Results

### Study population: baseline characteristics

**Figure 1:**
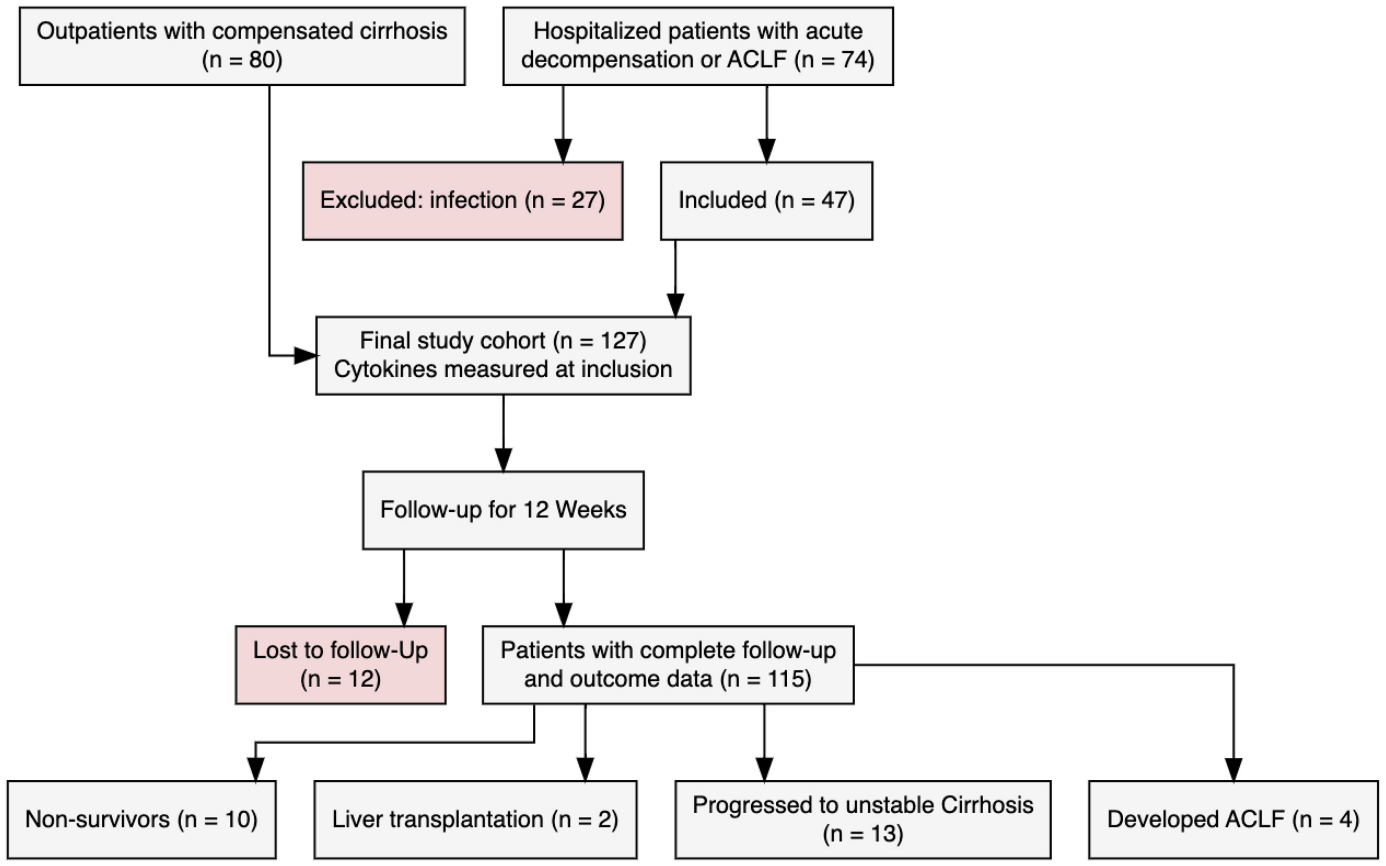
Diagram plot.

A total of 127 patients were included in this study. Of these, 80 patients (63%) had compensated cirrhosis, 29 (23%) had acute decompensation, and 18 (14%) had ACLF at baseline (**Figure 1**). Within the ACLF group, 72% were classified as ACLF grade 1, and 28% as grade 2 and 3 at baseline. Baseline characteristics of all patients are summarized in **Tables 1A**, **B**. Patients’ basic characteristics significantly differed across cirrhosis stages. As expected, MELD score was notably higher in the ACLF group (26.5 ± 6.9) than in the acute decompensation (15.4 ± 5.4) and compensated cirrhosis (14.2 ± 7.2) groups (p<0.001). Age distribution was similar across groups (p=0.500). INR, bilirubin, creatinine, and CRP levels increased significantly from compensated cirrhosis to ACLF (p < 0.05), whereas hemoglobin and albumin levels decreased significantly with advancing severity (both p<0.001). Ascites was significantly more prevalent in acute decompensation (93%) and ACLF (100%) compared to compensated cirrhosis (24%, p<0.001). The prevalence of apparent hepatic encephalopathy also increased significantly from compensated cirrhosis to acute decompensation (24.1%) and ACLF (38.9%, p<0.001). There were no significant differences in gender and causes of cirrhosis across groups.

**Table 1A:** Characteristics of the patients according to phenotypes at enrollment.

Variable	Compensated cirrhosis (mean ± SD)	Acute decompensation (mean ± SD)	ACLF (mean ± SD)	P-value
MELD Score	14.19 ± 7.18	15.41 ± 5.43	26.50 ± 6.85	<0.001
Age (years)	55.09 ± 10.99	54.10 ± 12.38	58.11 ± 12.60	0.500
Leukocytes (10^9^/L)	6.79 ± 5.60	5.25 ± 3.24	8.38 ± 6.30	0.168
INR	1.25 ± 0.23	1.43 ± 0.32	1.68 ± 0.56	0.001
Bilirubin (mg/dL)	2.12 ± 1.89	4.19 ± 5.97	6.73 ± 9.83	0.021
Creatinine (mg/dL)	0.99 ± 0.70	1.00 ± 0.40	2.11 ± 1.05	<0.001
Hemoglobin (g/dL)	12.16 ± 2.05	9.56 ± 2.51	8.36 ± 1.52	<0.001
Platelets (10^9^/L)	123.06 ± 81.85	97.41 ± 55.53	100.06 ± 59.81	0.315
Sodium (mmol/L)	138.72 ± 3.92	138.34 ± 5.58	136.39 ± 5.54	0.179
Potassium (mmol/L)	4.18 ± 0.51	3.92 ± 0.54	4.06 ± 0.67	0.098
ALT (GPT, U/L)	50.29 ± 67.28	62.11 ± 90.27	33.12 ± 16.99	0.360
AST (GOT, U/L)	61.69 ± 61.19	87.86 ± 130.50	65.71 ± 42.16	0.410
γ-GT (U/L)	137.34 ± 172.22	112.48 ± 119.55	96.61 ± 97.70	0.209
Alkaline Phosphatase (U/L)	172.56 ± 155.80	172.79 ± 123.18	113.50 ± 49.17	0.047
Albumin (g/dL)	3.63 ± 0.57	2.98 ± 0.55	3.09 ± 0.57	<0.001
C-reactive Protein (mg/dL)	0.40 ± 0.60	2.46 ± 2.58	2.71 ± 2.41	<0.001

**Table 1B:** 

Variable	Category	Compensated cirrhosis (n=80)	Acute decompensation (n=29)	ACLF (n=18)	P-value
Sex	Male	42 (53%)	20 (69%)	13 (72%)	0.143
Cause of Cirrhosis	ASH	11 (14%)	6 (21%)	4 (22%)	0.543
	MASH	39 (49%)	11 (38%)	10 (56%)	–
	Other	30 (37%)	12 (41%)	4 (22%)	–
Non-Survivors	–	2 (3%)	5 (17.2%)	3 (16.7%)	0.007
Hepatic Encephalopathy	Present	0 (0.0%)	7 (24.1%)	7 (38.9%)	<0.001
HE Grade	0	80 (100%)	22 (75.9%)	11 (61.1%)	<0.001
	1	0 (0.0%)	3 (10.3%)	4 (22.2%)	–
	2	0 (0.0%)	4 (13.8%)	3 (16.7%)	–
Ascites	Present	19 (24.0%)	27 (93.1%)	18 (100%)	<0.001
ICA Grade	0	61 (76.0%)	2 (6.9%)	0 (0.0%)	<0.001
	1	12 (15.0%)	4 (13.8%)	1 (5.6%)	–
	2	4 (5.0%)	11 (37.9%)	8 (44.4%)	–
	3	3 (4.0%)	12 (41.4%)	9 (50.0%)	–
Renal Insufficiency	Present	0 (0.0%)	7 (24.1%)	17 (94.4%)	–

ACLF, Acute-on-Chronic Liver Failure; MELD, Model for End-Stage Liver Disease; INR, International Normalized Ratio; CRP, C-reactive protein; IL, Interleukin; ALT, Alanin-Aminotransferase, AST, Aspartat-Aminotransferase, γ-GT, Gamma-Glutamyltransferase; HE, hepatic encephalopathy; ICA, internation club of ascites; MASH, Metabolic Dysfunction-Associated Steatohepatitis; ASH, Alcoholic steatohepatitis.

### Study population: follow‐up until week 12

By the end of the 12-week follow-up period, 10 patients (8%) had died, 2 patients (2%) had undergone liver transplantation, and 12 patients (9%) were lost to follow-up. Of the remaining patients, 13 patients experienced a decompensating event and progressed to unstable decompensated cirrhosis, while 4 patients developed ACLF. A comparative overview of baseline characteristics and inflammatory marker profiles between patients who died within 12 weeks of study enrollment and those who survived is provided in **Tables 2A**, **B**. The mean MELD score was slightly higher in non-survivors (18.1 ± 8.8) compared to survivors (16.3 ± 8.1), but this difference was not statistically significant (*p* = 0.561). Age was nearly identical in both groups (54.9 ± 11.7 vs. 54.7 ± 13.9 years, *p* = 1.000). When comparing laboratory parameters, most liver function markers were not significantly different between groups. However, non-survivors had significantly lower hemoglobin levels (9.0 ± 2.2 g/dL vs. 11.4 ± 2.5 g/dL, *p* = 0.006), and CRP levels were significantly higher (2.7 ± 3.1 mg/dL vs. 1.0 ± 1.7 mg/dL, *p* = 0.015). There were also no major differences in sex distribution or the etiology of cirrhosis. Hepatic encephalopathy was numerically more frequent among non-survivors (30%) than survivors (10%) (*p* = 0.152) but did not reach statistical significance. In contrast, ascites was significantly more prevalent in non-survivors (90.0% vs. 46.7%, *p* = 0.022).

**Table 2A:** Clinical and laboratory parameters by survival status.

Variable	Survivor (mean ± SD)	Non-survivor (mean ± SD)	P-value
MELD Score	16.25 ± 8.14	18.10 ± 8.79	0.561
Age (years)	54.92 ± 11.70	54.70 ± 13.94	1.000
Leukocytes (10^9^/L)	5.35 ± 4.87	8.44 ± 5.43	0.228
INR	1.33 ± 0.33	1.54 ± 0.38	0.087
Bilirubin (mg/dL)	3.31 ± 5.27	4.23 ± 5.81	0.306
Creatinine (mg/dL)	1.13 ± 0.81	1.07 ± 0.54	0.952
Hemoglobin (g/dL)	11.35 ± 2.46	9.04 ± 2.15	0.006
Platelets (10^9^/L)	116.45 ± 74.61	129.90 ± 89.50	0.913
Sodium (mmol/L)	138.52 ± 4.03	135.40 ± 8.22	0.146
Potassium (mmol/L)	4.13 ± 0.55	3.83 ± 0.59	0.170
ALT (GPT, U/L)	52.70 ± 74.51	45.50 ± 33.95	0.869
AST (GOT, U/L)	70.06 ± 87.60	65.10 ± 26.86	0.336
γ-GT (U/L)	129.71 ± 160.01	96.00 ± 91.86	0.388
Alkaline Phosphatase (U/L)	167.26 ± 143.17	194.00 ± 165.73	0.751
Albumin (g/dL)	3.45 ± 0.61	3.00 ± 0.86	0.092
CRP (mg/dL)	1.02 ± 1.67	2.69 ± 3.08	0.015

**Table 2B:** 

Variable	Category	Survivor (n=103)	Non-survivor (n=10)	P-value
Sex	Male	61 (59.2%)	6 (60.0%)	1.000
Cause of Cirrhosis	ASH	19 (18.4%)	1 (10.0%)	0.301
	MASH	49 (47.6%)	3 (30.0%)	–
	Other	35 (34.0%)	6 (60.0%)	–
Hepatic Encephalopathy	Present	10 (9.7%)	3 (30.0%)	0.152
HE Grade	0	93 (90.3%)	7 (70.0%)	0.111
	1	5 (4.9%)	2 (20.0%)	–
	2	5 (4.9%)	1 (10.0%)	–
Ascites	Present	49 (47.6%)	9 (90.0%)	0.022
ICA Grade	0	57 (55.3%)	1 (10.0%)	0.018
	1	14 (13.6%)	1 (10.0%)	–
	2	17 (16.5%)	3 (30.0%)	–
	3	15 (14.6%)	5 (50.0%)	–
Renal Insufficiency	Present	16 (15.5%)	5 (50.0%)	1.000

ACLF, Acute-on-Chronic Liver Failure; MELD, Model for End-Stage Liver Disease; INR, International Normalized Ratio; CRP, C-reactive protein; IL, Interleukin. ALT, Alanin-Aminotransferase; AST, Aspartat-Aminotransferase; γ-GT, Gamma-Glutamyltransferase; HE, hepatic encephalopathy; ICA, internation club of ascites; MASH, Metabolic Dysfunction-Associated Steatohepatitis; ASH, Alcoholic steatohepatitis.

### Unadjusted analysis of cytokines across disease stages

**Figure 2:**
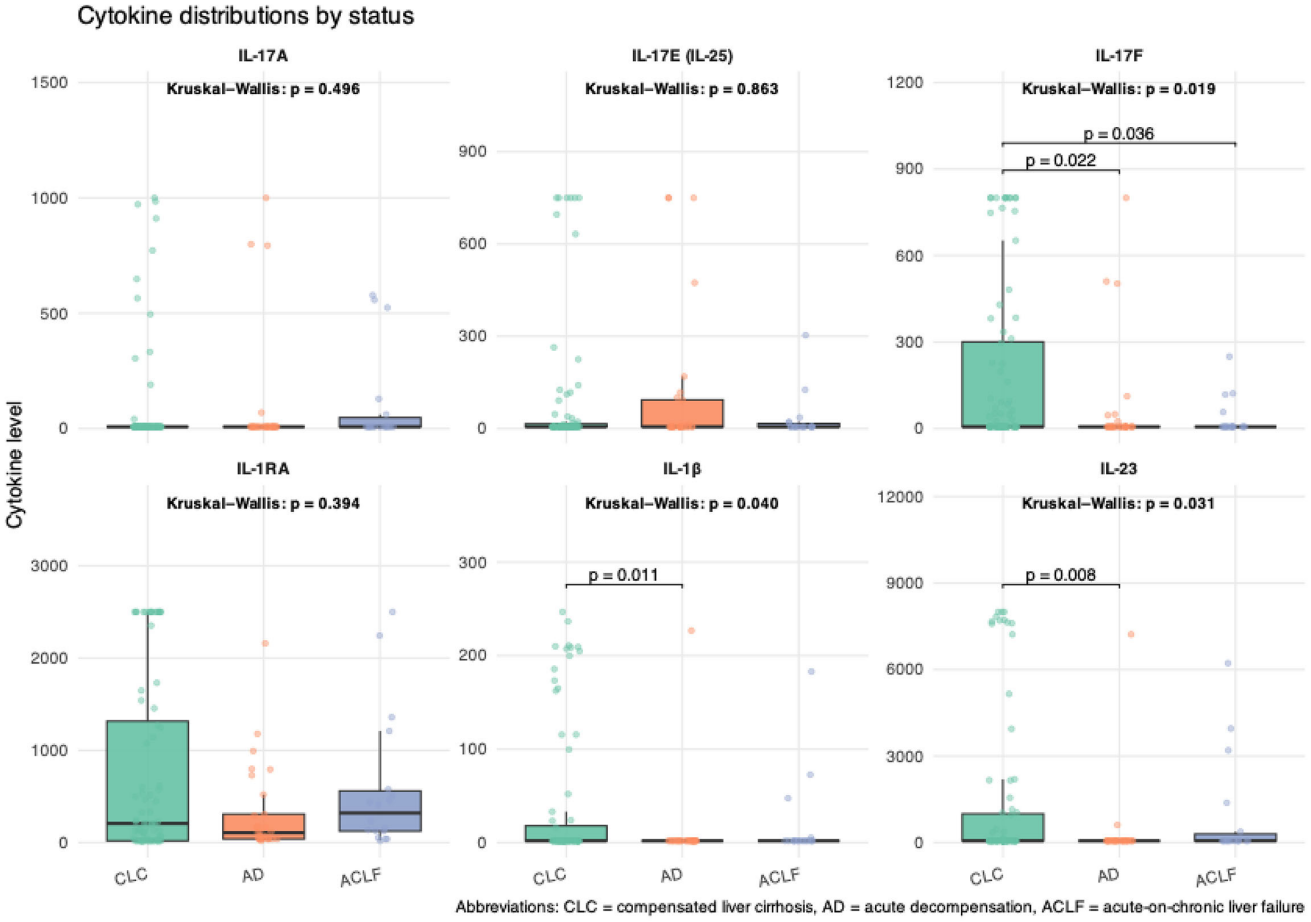
Box plots illustrating the distribution of cytokine levels across stages of liver disease. Cytokine concentration (pg/mL).

Cytokine levels were analyzed at different stages of liver disease, encompassing both conventional classifications (compensated cirrhosis, acute decompensation, and ACLF) (**Tables 3A**, **B**). IL-17F showed a statistically significant overall difference (*p* = 0.019). *Post-hoc* testing indicated that IL-17F levels were significantly higher in patients with compensated cirrhosis compared to those with acute decompensation (*p* = 0.022) and ACLF (*p* = 0.036). In a similar pattern, IL-23 levels were significantly higher in compensated cirrhosis (1405.7 ± 2691.0 pg/mL) compared to acute decompensation (328.4 ± 1329.7 pg/mL) (p=0.031). IL-1β levels also differed significantly between groups (*p* = 0.040), with a marked decrease observed from compensated to acutely decompensated stages. In contrast, levels of IL-17A, IL-17E, and IL-1RA levels did not show significant differences (**Figure 2** **).**

**Table 3A:** Cytokine levels by status of cirrhosis.

Marker	Compensated cirrhosis (mean ± SD)	Acute decompensation (mean ± SD)	ACLF (mean ± SD)	P-value
IL-17A	96.83 ± 247.01	98.49 ± 266.76	108.40 ± 207.16	0.496
IL-17F	192.31 ± 293.66	75.27 ± 189.69	35.22 ± 65.32	0.019
IL-17E	92.85 ± 218.40	114.96 ± 238.37	32.39 ± 73.44	0.863
IL-1RA	745.33 ± 966.27	319.22 ± 481.61	590.71 ± 751.78	0.394
IL-23	1405.67 ± 2690.97	328.36 ± 1329.70	886.19 ± 1758.47	0.031
IL-1β	40.17 ± 75.32	9.70 ± 41.74	18.64 ± 45.25	0.040

**Table 3B:** 

Marker	Comparison	P-value
IL-17F	Comp. cirrhosis vs. Acute Decomp.	0.022
	Comp. cirrhosis vs. ACLF	0.036
IL-1β	Comp. cirrhosis vs. Acute Decomp.	0.011
IL-23	Comp. cirrhosis vs. Acute Decomp.	0.008
Other comparisons		>0.05

Post-Hoc

### Multinomial regression analysis and association with clinical phenotypes

Next, we conducted multinomial logistic regression analyses. Log-transformation of interleukin levels was performed to normalize their distribution and reduce skewness. Univariable associations are summarized in **Table 4**. In the unadjusted model, several parameters were significantly associated with transitions from compensated cirrhosis to more advanced disease states. Elevated MELD scores, INR, bilirubin, and creatinine levels were positively associated with both progression to AD and ACLF. Conversely, higher levels of hemoglobin and albumin levels were negatively associated with disease progression. Regarding cytokines, higher levels of IL-17F were significantly associated with reduced odds of progression to both AD (OR = 0.726; 95% CI = 0.548–0.963; p = 0.026) and to ACLF (OR = 0.689; 95% CI = 0.477–0.995; p = 0.047). Similarly, IL-23 (OR = 0.613; 95% CI = 0.399–0.941; p = 0.025) and IL-1β (OR = 0.561; 95% CI = 0.327–0.960; p = 0.035) were inversely associated with progression to AD; however, no significant association was observed for progression to ACLF. Other interleukins, including IL-17A, IL-17E, and IL-1RA, showed no significant associations in the univariable analysis (p > 0.1).

**Table 4:** Sociation between levels of circulating cytokines and odds of getting AD, or ACLF using unadjusted multinomial logistic regression.

Variable	Transition	OR	95% CI	*P*-value
MELD Score	Comp. cirrhosis → Acute decompensation	1.031	0.965 – 1.101	0.368
	Comp. cirrhosis → ACLF	1.206	1.116 – 1.303	<0.001
INR	Comp. cirrhosis → Acute decompensation	8.794	1.907 – 40.557	0.005
	Comp. cirrhosis → ACLF	35.204	6.344 – 195.349	<0.001
Bilirubin	Comp. cirrhosis → Acute decompensation	1.197	1.016 – 1.411	0.032
	Comp. cirrhosis → ACLF	1.249	1.057 – 1.477	0.009
Creatinine	Comp. cirrhosis → Acute decompensation	1.071	0.432 – 2.652	0.883
	Comp. cirrhosis → ACLF	4.565	2.071 – 10.065	<0.001
Hemoglobin	Comp. cirrhosis → Acute decompensation	0.590	0.468 – 0.743	<0.001
	Comp. cirrhosis → ACLF	0.424	0.299 – 0.602	<0.001
Sodium	Comp. cirrhosis → Acute decompensation	0.982	0.893 – 1.080	0.713
	Comp. cirrhosis → ACLF	0.908	0.818 – 1.008	0.070
Potassium	Comp. cirrhosis → Acute decompensation	0.388	0.163 – 0.924	0.033
	Comp. cirrhosis → ACLF	0.654	0.245 – 1.745	0.396
Albumin	Comp. cirrhosis → Acute decompensation	0.130	0.051 – 0.330	<0.001
	Comp. cirrhosis → ACLF	0.184	0.067 – 0.504	0.001
IL-23 (log)	Comp. cirrhosis → Acute decompensation	0.613	0.399 – 0.941	0.025
	Comp. cirrhosis → ACLF	0.939	0.700 – 1.260	0.676
IL-17A (log)	Comp. cirrhosis → Acute decompensation	0.975	0.719 – 1.322	0.873
	Comp. cirrhosis → ACLF	1.150	0.846 – 1.562	0.372
IL-17F (log)	Comp. cirrhosis → Acute decompensation	0.726	0.548 – 0.963	0.026
	Comp. cirrhosis → ACLF	0.689	0.477 – 0.995	0.047
IL-17E (log)	Comp. cirrhosis → Acute decompensation	1.080	0.834 – 1.399	0.560
	Comp. cirrhosis → ACLF	0.884	0.604 – 1.293	0.525
IL-1β (log)	Comp. cirrhosis → Acute decompensation	0.561	0.327 – 0.960	0.035
	Comp. cirrhosis → ACLF	0.858	0.599 – 1.229	0.402
IL-1RA (log)	Comp. cirrhosis → Acute decompensation	0.854	0.657 – 1.108	0.235
	Comp. cirrhosis → ACLF	1.090	0.803 – 1.480	0.581
CRP	Comp. cirrhosis → Acute decompensation	2.04	0.91 – 3.12	0.294
	Comp. cirrhosis → ACLF	2.12	0.78– 3.26	0.294
Age	Comp. cirrhosis → Acute decompensation	0.993	0.957 – 1.029	0.695
	Comp. cirrhosis → ACLF	1.026	0.977 – 1.077	0.309
Leukocytes	Comp. cirrhosis → Acute decompensation	0.939	0.815 – 1.081	0.381
	Comp. cirrhosis → ACLF	0.999	0.995 – 1.004	0.761
Platelets	Comp. cirrhosis → Acute decompensation	0.995	0.988 – 1.002	0.126
	Comp. cirrhosis → ACLF	0.995	0.987 – 1.003	0.261

OR, Odds Ratio; CI, Confidence Interval; ACLF, Acute-on-Chronic Liver Failure; MELD, Model for End-Stage Liver Disease; INR, International Normalized Ratio; CRP, C-reactive protein; IL, Interleukin.

**Figure 3:**
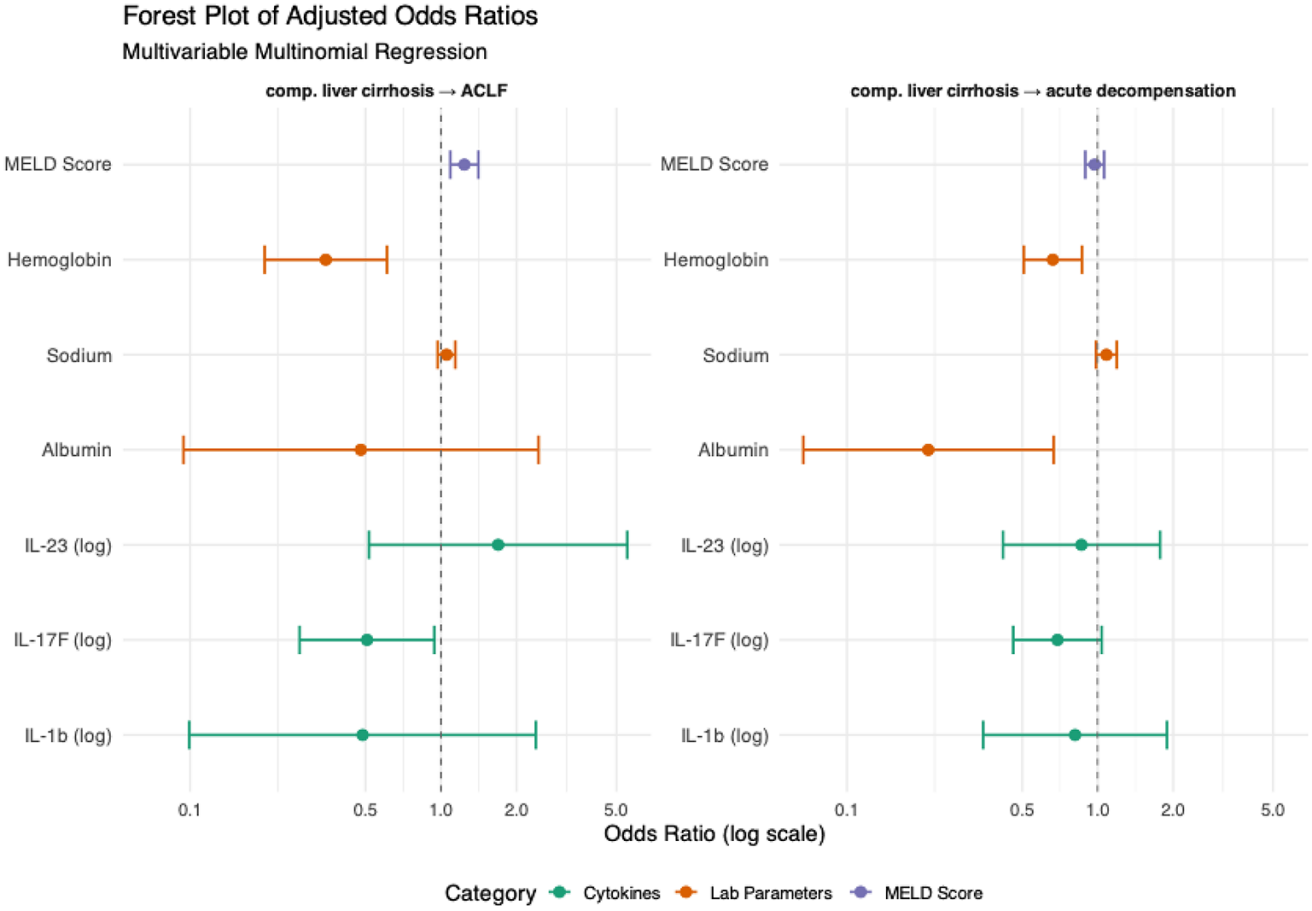
Multinomial regression analysis and association associations between circulating cytokines and clinical phenotypes. Using patients with compensated cirrhosis as the reference group, we assessed the adjusted associations between disease stages (acute decompensation or ACLF) and circulating inflammatory markers through multivariable multinomial logistic regression analysis. Covariates included in the model were selected based on a significance threshold of p < 0.1 in unadjusted univariable analyses and encompassed clinically relevant variables. Hemoglobin (g/dL), Sodium (mmol/L), Albumin (g/dL).

In the next step, we proceeded with the adjusted aforementioned multivariable model. As shown in **Table 5**, the transition from compensated cirrhosis to acute decompensation was independently associated with lower hemoglobin levels (OR = 0.662; 95% CI: 0.507–0.865; p = 0.002) and lower albumin levels (OR = 0.211; 95% CI: 0.067–0.667; p = 0.008). IL-17F showed a non-significant trend toward inverse association with acute decompensation (OR = 0.691; 95% CI: 0.460–1.037; p = 0.074). Similarly, the transition to ACLF showed a strong inverse association with hemoglobin (OR = 0.347; 95% CI: 0.198–0.609; p < 0.001), suggesting that anemia is a robust predictor of advanced disease progression. Notably, IL-17F remained significantly associated with a lower risk of ACLF even after adjustment (OR = 0.507; 95% CI: 0.273–0.941; p = 0.031), supporting its potential role as a protective immunoregulatory factor. In contrast, IL-23 and IL-1β did not retain statistical significance in the adjusted model, indicating that their associations may be context-dependent or confounded by other variables. As expected, the MELD score was as a strong independent predictor of progression to ACLF (OR = 1.239; 95% CI: 1.089–1.410; p = 0.001), while albumin and sodium lost significance after adjustment (**Figure 3**).

**Table 5:** Association between levels of circulating cytokines and odds of getting AD or ACLF using adjusted multinomial logistic regression.

Variable	Transition	Odds ratio (OR)	95% Confidence interval	P-value
(Intercept)	Comp. cirrhosis → Acute decompensation	1.33	[0 – 260,317.93]	0.964
MELD Score		0.97	[0.89 – 1.06]	0.523
Hemoglobin		0.66	0.51 – 0.87	0.002
Sodium		1.08	0.99 – 1.19	0.100
Albumin		0.21	0.07 – 0.67	0.008
IL-23 (log)		0.86	0.42 – 1.77	0.685
IL-17F (log)		0.69	0.46 – 1.04	0.074
IL-1β (log)		0.81	0.35 – 1.89	0.628
(Intercept)	Comp. cirrhosis → ACLF	1.55	0.001 – 4,440.73	0.914
MELD Score		1.24	1.09 – 1.41	0.001
Hemoglobin		0.35	0.20 – 0.61	<0.001
Sodium		1.05	0.97 – 1.14	0.218
Albumin		0.48	0.09 – 2.45	0.377
IL-23 (log)		1.69	0.52 – 5.53	0.386
IL-17F (log)		0.51	0.27 – 0.94	0.031
IL-1β (log)		0.49	0.10 – 2.39	0.375

OR, Odds Ratio; CI, Confidence Interval; ACLF, Acute-on-Chronic Liver Failure; MELD, Model for End-Stage Liver Disease; IL, Interleukin.

### Ordinal regression analysis

Univariable ordinal regression was also performed to identify predictors associated with increasing severity of liver disease as defined by clinical stage (**Table 6**). Markers of liver function and systemic inflammation showed strong associations. INR (OR = 22.77; 95% CI: 6.8–87.81; *p* = 5.83 × 10⁻^8^), CRP (OR = 1.74; 95% CI:1.38–2.30; *p* = 5.42 × 10⁻^8^), and bilirubin levels (OR = 1.12, 95% CI: [1.05–1.23], *p* = 9.42 × 10⁻^4^) were all significantly associated with more advanced stages. Likewise, a higher MELD score (OR = 1.13; 95% CI 1.07–1.20; *p* = 6.19 × 10⁻⁷) and serum creatinine level (OR = 3.65; 95% CI:2.01–7.15; *p* = 7.13 × 10⁻^6^) were significant predictors. Conversely, protective associations were observed for hemoglobin (OR = 0.51; 95% CI: 0.40–0.62;p = 1.66 × 10⁻¹^4^), albumin (OR = 0.18; 95% CI: 0.09–0.35; *p* = 1.23 × 10⁻^9^), and serum sodium levels (OR = 0.91; 95% CI: 0.84–0.98; *p* = 0.014). Cytokine markers also revealed significant associations: IL-17F was inversely associated with disease severity (OR = 0.71; 95% CI: 0.55–0.90; *p* = 0.003). IL-1β (OR = 0.72; *p* = 0.018) and IL-23 (OR = 0.78; *p* = 0.023) also showed significant negative associations. Other variables such as potassium level (*p* = 0.059), age (*p* = 0.409), and various cytokines including IL-17A, IL-17E, and IL-1RA, were not significantly associated with disease stage in this univariable analysis (**Supplementary Figure 1**).

**Table 6:** Univariable ordinal logistic regression.

Predictor	Odds ratio (OR)	95% Confidence interval	P-value
Hemoglobin (g/dL)	0.51	0.40 – 0.62	<0.0001
Albumin (g/dL)	0.18	0.09 – 0.35	<0.0001
CRP (mg/dL)	1.74	1.38 – 2.30	<0.0001
INR	22.77	6.80 – 87.81	<0.0001
MELD Score	1.13	1.07 – 1.20	<0.0001
Creatinine (mg/dL)	3.65	2.01 – 7.15	<0.0001
Bilirubin (mg/dL)	1.12	1.05 – 1.23	0.0009
IL-17F (log)	0.71	0.55 – 0.90	0.0032
Sodium (mmol/L)	0.91	0.84 – 0.98	0.0141
IL-1β (log)	0.72	0.51 – 0.95	0.0184
IL-23 (log)	0.78	0.60 – 0.99	0.0233
Potassium (mmol/L)	0.52	0.25 – 1.03	0.0592
Platelets (10^9^/L)	1.00	0.99 – 1.00	0.183
Leukocytes (10^9^/L)	1.00	0.99 – 1.00	0.352
Age (years)	1.01	0.98 – 1.05	0.409
IL-17E (log)	0.92	0.73 – 1.14	0.463
IL-17A (log)	1.07	0.84 – 1.34	0.582
IL-1RA (log)	0.95	0.76 – 1.18	0.629

ACLF, Acute-on-Chronic Liver Failure; MELD, Model for End-Stage Liver Disease; INR, International Normalized Ratio; CRP, C-reactive protein; IL, Interleukin.

**Figure 4:**
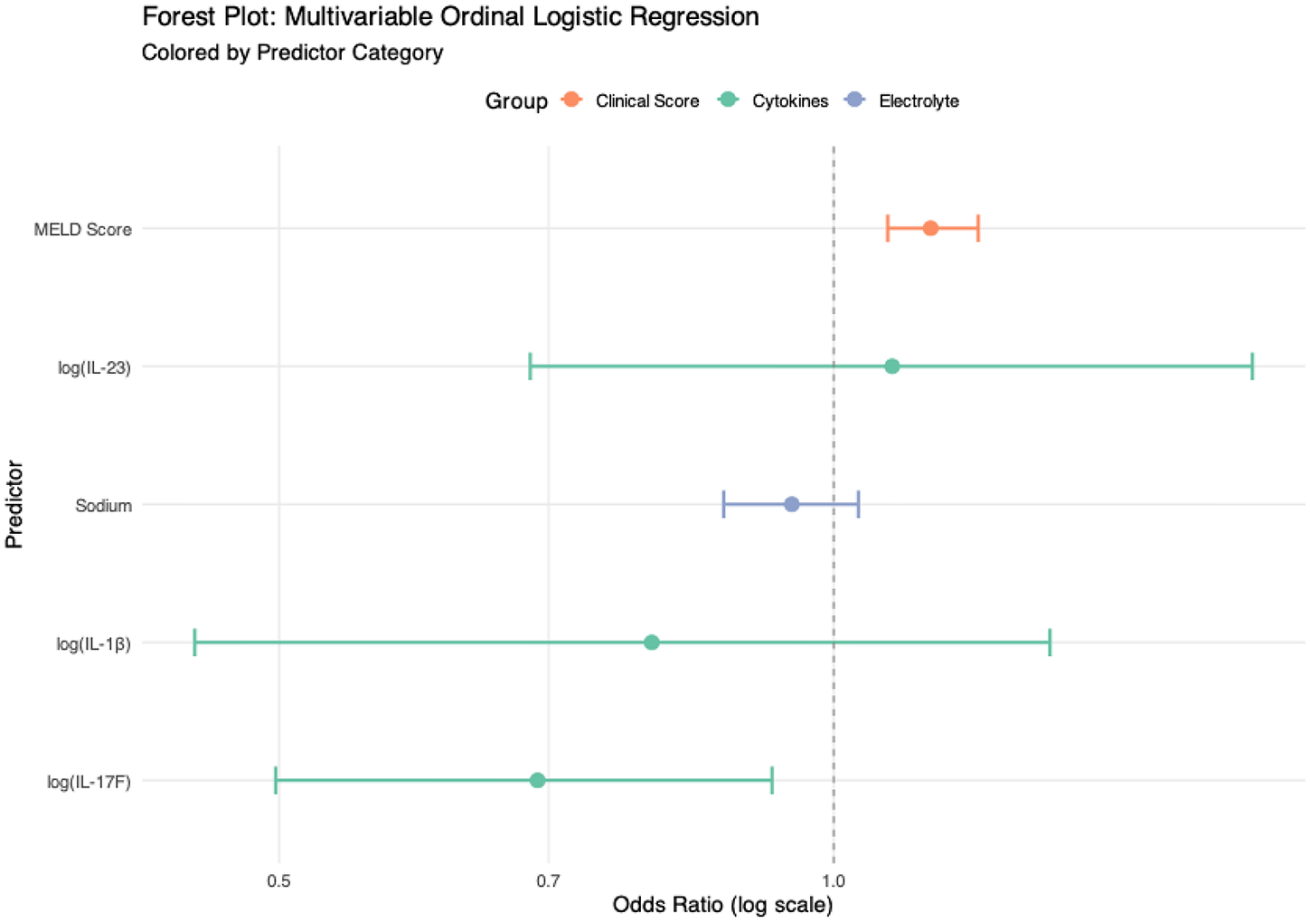
Multivariablel ordinal analysis and associations between circulating cytokines and clinical phenotypes. Using patients with compensated cirrhosis as the reference group, we assessed the adjusted associations between disease stages (acute decompensation or ACLF) and circulating inflammatory markers through multivariable ordinal logistic regression analysis. Sodium (mmol/L).

In the multivariable ordinal regression model, MELD score continued to show a strong positive association (OR = 1.13; *p* < 0.001), while IL-17F retained its inverse relationship (OR = 0.69; 95% CI: 0.50–0.93; *p* = 0.018).IL-1β, IL-23, and sodium, however, lost significance after adjustment (**Figure 4**; **Table 7**).

**Table 7:** Multivariable ordinal logistic regression.

Predictor	Odds ratio (OR)	95% Confidence interval	P-value
MELD Score	1.13	1.07 – 1.20	<0.001
IL-17F (log)	0.69	0.50 – 0.93	0.018
Sodium (mmol/L)	0.95	0.87 – 1.03	0.217
IL-1β (log)	0.80	0.45 – 1.31	0.392
IL-23 (log)	1.08	0.68 – 1.69	0.747

MELD, Model for End-Stage Liver Disease; IL, Interleukin.

### Relationship between serum cytokines and survival

**Figure 5:**
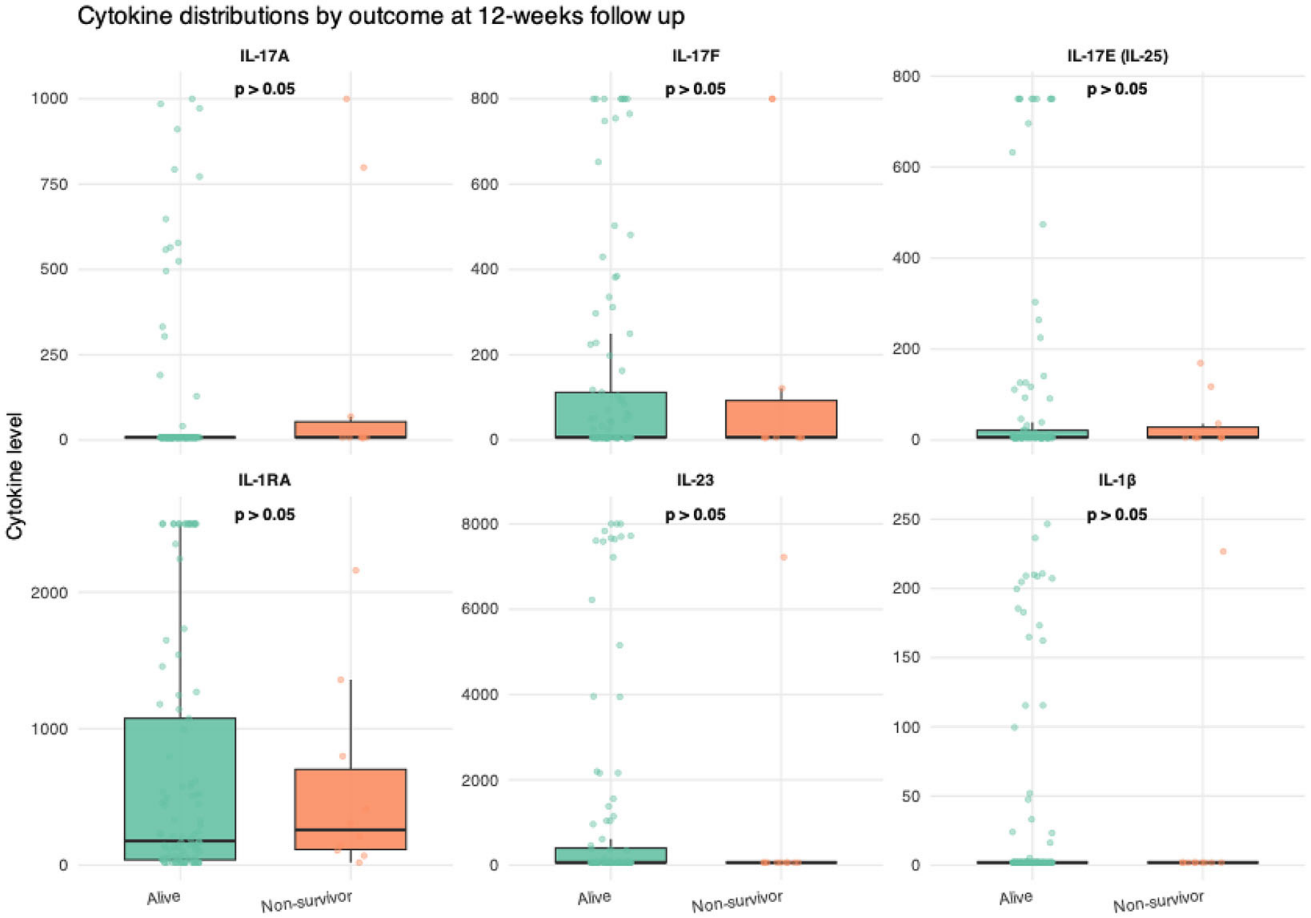
Box plots illustrating the distribution of cytokine levels by survival status. Cytokine concentration (pg/mL).

Cytokine concentrations were compared between survivors and non-survivors at the 12-week follow-up. No cytokine showed a significant association with 12-week mortality in either unadjusted or adjusted analyses. IL-17A levels were numerically higher in non-survivors (192.3 ± 376.2 pg/mL) compared with survivors (99.9 ± 242.0 pg/mL); however, this difference did not reach statistical significance (p = 0.244). Likewise, no significant differences were observed for IL-17F (p = 0.800), IL-17E (p = 0.922), IL-23 (p = 0.235), IL-1β (p = 0.429), or IL-1RA (p = 0.727) (**Figure 5**; **Table 8**).

**Table 8:** Cytokine levels by survival status.

Marker	Survivor (mean ± SD)	Non-survivor (mean ± SD)	P-value
IL-17A	99.85 ± 241.99	192.27 ± 376.21	0.244
IL-17E	102.93 ± 227.35	36.38 ± 58.49	0.922
IL-17F	143.02 ± 253.66	176.54 ± 330.56	0.800
IL-1RA	678.33 ± 907.47	558.12 ± 697.37	0.727
IL-1β	33.26 ± 69.12	24.43 ± 71.09	0.429
IL-23	1200.48 ± 2469.04	778.41 ± 2263.92	0.235

Due to the limited number of mortality events, logistic regression was employed in place of Cox proportional hazards modeling. In the unadjusted analyses, none of the examined cytokines were significantly associated with 12-week mortality, indicating limited prognostic value within this cohort (**Supplementary Table 1**).

### IL-23/IL-17 Axis Cytokines and Their Clinical Correlates

To further explore the relationship between cytokine expression and organ-specific complications of cirrhosis, we assessed levels of IL-23/IL-17 axis cytokines in relation to the presence of hepatic encephalopathy (HE) cascites, and renal failure. No statistically significant differences in cytokine concentrations were observed between patients with or without these complications. IL-17A levels were similar in patients with and without HE (87.3 ± 264.7 vs. 100.3 ± 243.3 pg/mL; p = 0.835), ascites (96.4 ± 241.2 vs. 101.4 ± 250.3 pg/mL; p = 0.917), and renal failure (120.4 ± 251.6 vs. 93.3 ± 244.7 pg/mL; p = 0.523). Likewise, no significant differences were found for IL-17F, IL-17E, IL-1β, IL-1RA, and IL-23 across these clinical subgroups (all p > 0.1; **Table 9**).

**Table 9:** Cytokine levels by liver cirrhosis complications.

Cytokine	Renal insufficiency: No	Renal insufficiency: Yes	P-value
	(Mean ± SD)	(Mean ± SD)	
IL-17A	93.34 ± 244.68	120.43 ± 251.57	0.523
IL-17E	103.91 ± 231.49	42.31 ± 103.05	0.506
IL-17F	155.68 ± 276.19	106.02 ± 192.48	0.865
IL-1RA	591.51 ± 865.45	720.51 ± 867.88	0.098
IL-1β	35.25 ± 72.21	10.74 ± 35.61	0.115
IL-23	1183.77 ± 2483.23	513.10 ± 1313.18	0.244

Cytokine	Hepatic encephalopathy: No	Hepatic encephalopathy: Yes	P-value
	(Mean ± SD)	(Mean ± SD)	
IL-17A	100.28 ± 243.33	87.32 ± 264.66	0.835
IL-17E	98.49 ± 220.30	15.39 ± 24.11	0.394
IL-17F	148.73 ± 263.98	99.64 ± 213.91	0.791
IL-1RA	663.12 ± 888.40	327.41 ± 551.45	0.659
IL-1β	31.26 ± 67.19	21.26 ± 60.37	0.570
IL-23	1112.44 ± 2389.54	852.06 ± 2106.65	0.356

Cytokine	Ascites: No	Ascites: Yes	P-value
	(Mean ± SD)	(Mean ± SD)	
IL-17A	101.44 ± 250.27	96.38 ± 241.17	0.917
IL-17E	90.41 ± 213.28	88.30 ± 207.45	0.908
IL-17F	162.39 ± 276.63	125.13 ± 241.01	0.723
IL-1RA	647.16 ± 900.76	606.04 ± 831.11	0.944
IL-1β	38.51 ± 73.52	22.19 ± 58.11	0.136
IL-23	1478.48 ± 2771.28	712.84 ± 1824.69	0.193

Subsequently, we examined correlations between interleukin concentrations and routine laboratory parameters. None of the interleukins showed a strong correlation with standard clinical markers, suggesting that their expression may reflect distinct immunological processes and could serve as independent biomarkers in the context of cirrhosis (**Supplementary Figure 2**).

To further explore the kinetics of interleukin regulation in advanced liver disease, we investigated the correlations between the circulating levels of individual cytokines. The strongest correlation was observed between IL-1β and IL-23 (r = 0.797, p < 0.001), suggesting a coordinated upregulation of these innate immune mediators. IL-23 also showed a high correlation with IL-1RA (r = 0.718). IL-17F correlated weakly with IL-17A (r = 0.386), IL-23 (r = 0.466), and IL-1β (r = 0.470). These patterns support the hypothesis that IL-17F may be functionally linked to IL-23–driven responses but regulated independently of IL-17A. (**Supplementary Figure 3**).

## Discussion

In the present study, we analyzed six cytokines of the IL-23/IL-17 signaling axis in the patients from two independent cohorts with cirrhosis in the absence of active infection. A total of 127 patients were included, comprising 80 patients with compensated cirrhosis, 29 with AD, and 18 with ACLF.

Our study demonstrated that concentrations of IL-17F, IL-23 and IL-1b were significantly lower in patients with acute decompensation and ACLF compared to those with compensated cirrhosis, indicating a progressive decline with increasing disease severity. Unadjusted ordinal and multinomial regression analyses confirmed these findings. More importantly, adjusted analyses demonstrated that the progressive decline in IL-17F levels with increasingly severe clinical phenotypes of acute decompensation and ACLF was independent of other confounding factors.

Despite the exploratory nature of our analysis, none of the investigated cytokines were significantly associated with 12-week mortality. This is likely due to the small number of deaths (n = 10) and the resulting lack of statistical power. Consequently, the following discussion focuses on the potential role of IL-17F in cirrhosis progression, as its association with disease severity remained significant even after adjustment for confounding factors.

IL-23 and IL-17 are key pro-inflammatory mediators of the IL-23/IL-17 signaling axis (29). IL-1β, a regulator of early inflammation, is produced through the activation of inflammasome complexes, most notably the NLRP3 inflammasome, within macrophages and liver-resident Kupffer cells. IL-1β promotes the differentiation of naïve CD4^+^; T cells into Th17 cells and induces expression of the IL-23 receptor, thereby enhancing responsiveness to IL-23 (30, 31). IL-23, on the other hand, is primarily secreted by dendritic cells and macrophages, stabilizes the Th17 phenotype and supports its proliferation and survival (29). Importantly, Th17 cells are the best-known producers of IL-17A (32). While IL-17A is the most extensively studied member of the IL-17 cytokine family, the family includes five additional members, among which IL-17F holds particular interest. Its function is closely related to that of IL-17A, owing in part to their high amino acid sequence homology of approximately 50% (29, 33). Both IL-17A and IL-17F exert their effects by binding to a heterodimeric receptor complex composed of IL-17 receptor A (IL-17RA) and IL-17 receptor C (IL-17RC) on target cells, thereby initiating multiple intracellular signaling cascades (34, 35).

Despite their shared classification as pro-inflammatory Th17 cytokines, our findings raise a mechanistic question: how can the observed decline in IL-17F levels be reconciled with advancing disease severity, while IL-17A — though not statistically significant — shows a trend toward increased levels in more advanced stages?

A study investigating the mechanistic differences between IL-17A and IL-17F in the context of liver disease, using transcriptional network analysis, revealed distinct pathway activation profiles between the two cytokines (36). Notably, NFκB signaling was more strongly enriched among IL-17F–modulated differentially expressed genes, suggesting that IL-17A and IL-17F differ in the extent to which they activate the NFκB pathway (36). In this context, it is well established that activation of the NF-κB pathway induces the expression of pro-survival factors such as FLIP and the anti-apoptotic gene Bcl-2 (37). FLIP, in particular, can directly inhibit the activation of caspase-8, thereby blocking the intrinsic apoptotic pathway. Moreover, TNF-α can trigger NF-κB activation via the IKK complex following its binding to TNF receptors, highlighting the crucial role of NF-κB signaling in promoting cell survival and preventing apoptosis (38). In our cohort, we observed a decline in circulating IL-17F levels in advanced stages of cirrhosis. While the precise functional consequences of this observation remain to be determined, we hypothesize that reduced IL-17F expression may reflect a downregulation of protective NF-κB–mediated signaling pathways. This, in turn, could impair pro-survival mechanisms, potentially contributing to hepatocyte apoptosis, aggravated liver injury, and accelerated disease progression. These interpretations, however, are speculative and warrant future experimental validation in studies directly investigating IL-17F-mediated NF-κB signaling in cirrhosis.

Another layer of complexity is that studies have demonstrated that IL-17A and IL-17F differ in their tissue distribution and receptor-binding affinities (39, 40). Moreover, since serum levels of IL-17A and IL-17E — both typically secreted from Th17 cells — showed only weak correlation with IL-17F expression, it is likely that cell types other than Th17 cells contribute significantly to IL-17F production. A study of hepatitis C patients with liver fibrosis and hepatocellular carcinoma demonstrated that IL-17F expression was predominantly localized to hepatocytes, suggesting that serum IL-17F in these individuals may originate, at least in part, from hepatocyte-derived production (41). However, the specific stimuli that induce IL-17F expression in hepatocytes and other non-immune cell types, remain to be elucidated.

Our study has several important limitations that need to be addressed. Firstly, we were unable to fully control for specific confounders affecting immune status, including nutritional deficiencies or previous treatments such as beta-blockers, which are known to modulate systemic inflammation in advanced chronic liver disease. Although robust statistical methods were applied to mitigate these confounding effects, future studies should aim for comprehensive adjustments or exclusions for these variables. Secondly, assessing circulating cytokines in peripheral blood does not necessarily reflect inflammatory processes occurring directly within liver tissue. For instance, impaired renal function can reduce cytokine clearance, potentially leading to elevated concentrations in the bloodstream (42). Future research designs should therefore integrate direct sampling from liver tissue and portal vein blood. Moreover, in our study, inflammatory markers were measured exclusively at baseline, lacking subsequent serial assessments throughout disease progression. Future longitudinal studies employing repeated measures would significantly enhance our understanding of inflammation dynamics over time. Lastly, and importantly, the cytokines analyzed in this research can originate from multiple immune cell populations. An additional limitation of this study is the relatively small cohort size of AD/ACLF patients and the limited number of mortality events, which reduce the statistical power of the analyses and may affect the generalizability of the findings. In the absence of previous studies specifically addressing this question in ACLF, the present work should be regarded as exploratory, providing preliminary insights that require confirmation. Future research in larger, independent cohorts, together with mechanistic studies specifically investigating cytokine production and regulation by distinct immune cell populations, will be essential to further elucidate the underlying inflammatory mechanisms.

## Conclusion

Our findings demonstrate a selective decline in IL-17F levels with increasing cirrhosis severity, indicating a distinct immunoregulatory function that may diminish as the disease progresses. Unlike other IL-17 family cytokines, IL-17F showed a consistent inverse association with clinical deterioration and emerged as an independent predictor of disease progression from compensated cirrhosis to AD and ACLF. These results support its potential not only as a biomarker but also as a prognostic indicator in advanced liver disease. Further mechanistic studies are needed to clarify its precise role in cirrhosis pathophysiology.

## Data Availability

The original contributions presented in the study are included in the article/**Supplementary Material**. Further inquiries can be directed to the corresponding authors.

## Supplementary material

Supplementary Table 1 Univariable regression evaluating the association between circulating interleukin levels and 12-week mortality.

Supplementary Figure 1 Univariable Ordninal regression

Supplementary Figure 2 Spearman correlation matrix of baseline cytokines and standard clinical laboratory markers

Supplementary Figure 3 Spearman correlation between the cytokine levels

## References

[1] Albillos A Martin-Mateos R van der Merwe S Wiest R Jalan R Álvarez-Mon M. Cirrhosis-associated immune dysfunction. Nat Rev Gastroenterol Hepatology. (2022) 19:112–34. doi: 10.1038/s41575-021-00520-7, PMID: 34703031

[2] Arroyo V Moreau R Jalan R Ginès P Study E-CCC. Acute-on-chronic liver failure: a new syndrome that will re-classify cirrhosis. J hepatology. (2015) 62:S131–S43. doi: 10.1016/j.jhep.2014.11.045, PMID: 25920082

[3] Moreau R Jalan R Gines P Pavesi M Angeli P Cordoba J. Acute-on-chronic liver failure is a distinct syndrome that develops in patients with acute decompensation of cirrhosis. Gastroenterology. (2013) 144:1426–37. e9. doi: 10.1053/j.gastro.2013.02.042, PMID: 23474284

[4] Trebicka J Fernandez J Papp M Caraceni P Laleman W Gambino C. PREDICT identifies precipitating events associated with the clinical course of acutely decompensated cirrhosis. J hepatology. (2021) 74:1097–108. doi: 10.1016/j.jhep.2020.11.019, PMID: 33227350

[5] Monteiro S Grandt J Uschner FE Kimer N Madsen JL Schierwagen R. Differential inflammasome activation predisposes to acute-on-chronic liver failure in human and experimental cirrhosis with and without previous decompensation. Gut. (2021) 70:379–87. doi: 10.1136/gutjnl-2019-320170, PMID: 32241903 PMC7815638

[6] Albillos A Lario M Álvarez-Mon M. Cirrhosis-associated immune dysfunction: distinctive features and clinical relevance. J hepatology. (2014) 61:1385–96. doi: 10.1016/j.jhep.2014.08.010, PMID: 25135860

[7] Trebicka J Praktiknjo M Peiffer K-H Pascher A Schulz MS Uschner FE. Acute-on-chronic liver failure. Deutsches Ärzteblatt Int. (2025) 122. doi: 10.3238/arztebl.m2024.0255, PMID: 39773823 PMC12439201

[8] Rueschenbaum S Ciesek S Queck A Widera M Schwarzkopf K Brüne B. Dysregulated adaptive immunity is an early event in liver cirrhosis preceding acute-on-chronic liver failure. Front Immunol. (2021) 11:534731. doi: 10.3389/fimmu.2020.534731, PMID: 33574809 PMC7870861

[9] Lange CM Moreau R. Immunodysfunction in acute-on-chronic liver failure. Visceral Med. (2018) 34:276–82. doi: 10.1159/000488690, PMID: 30345285 PMC6189545

[10] Artru F Trovato F Morrison M Bernal W McPhail M. Liver transplantation for acute-on-chronic liver failure. Lancet Gastroenterol Hepatology. (2024) 9:564–76. doi: 10.1016/S2468-1253(23)00363-1, PMID: 38309288

[11] Moreau R Tonon M Krag A Angeli P Berenguer M Berzigotti A. EASL Clinical Practice Guidelines on acute-on-chronic liver failure. J hepatology. (2023) 79:461–91. doi: 10.1016/j.jhep.2023.04.021, PMID: 37364789

[12] Konstantis G Tsaousi G Pourzitaki C Kitsikidou E Magouliotis DE Wiener S. Efficacy of granulocyte colony-stimulating factor in acute on chronic liver failure: a systematic review and survival meta-analysis. J Clin Med. (2023) 12:6541. doi: 10.3390/jcm12206541, PMID: 37892679 PMC10607065

[13] Arroyo V Angeli P Moreau R Jalan R Clària J Trebicka J. The systemic inflammation hypothesis: towards a new paradigm of acute decompensation and multiorgan failure in cirrhosis. J hepatology. (2021) 74:670–85. doi: 10.1016/j.jhep.2020.11.048, PMID: 33301825

[14] Cao Z Yao Y Cai M Zhang C Liu Y Xin H. Blood markers for type-1, -2, and -3 inflammation are associated with severity of acutely decompensated cirrhosis. J Hepatol. (2024). doi: 10.1016/j.jhep.2024.10.028, PMID: 39490592

[15] Gaffen SL Jain R Garg AV Cua DJ. The IL-23–IL-17 immune axis: from mechanisms to therapeutic testing. Nat Rev Immunol. (2014) 14:585–600. doi: 10.1038/nri3707, PMID: 25145755 PMC4281037

[16] Liu T Li S Ying S Tang S Ding Y Li Y. The IL-23/IL-17 pathway in inflammatory skin diseases: from bench to bedside. Front Immunol. (2020) 11:594735. doi: 10.3389/fimmu.2020.594735, PMID: 33281823 PMC7705238

[17] Lubberts E. The IL-23–IL-17 axis in inflammatory arthritis. Nat Rev Rheumatol. (2015) 11:415–29. doi: 10.1038/nrrheum.2015.53, PMID: 25907700

[18] Sarra M Pallone F MacDonald TT Monteleone G. Il-23/il-17 axis in IBD. Inflammatory bowel diseases. (2010) 16:1808–13. doi: 10.1002/ibd.21248, PMID: 20222127

[19] Basho K Zoldan K Schultheiss M Bettinger D Globig A-M Bengsch B. IL-2 contributes to cirrhosis-associated immune dysfunction by impairing follicular T helper cells in advanced cirrhosis. J Hepatology. (2021) 74:649–60. doi: 10.1016/j.jhep.2020.10.012, PMID: 33211012

[20] Qian C Jiang T Zhang W Ren C Wang Q Qin Q. Increased IL-23 and IL-17 expression by peripheral blood cells of patients with primary biliary cirrhosis. Cytokine. (2013) 64:172–80. doi: 10.1016/j.cyto.2013.07.005, PMID: 23910013

[21] Vujovic A Isakovic AM Misirlic-Dencic S Juloski J Mirkovic M Cirkovic A. IL-23/IL-17 Axis in Chronic Hepatitis C and non-alcoholic steatohepatitis—new insight into immunohepatotoxicity of different Chronic Liver diseases. Int J Mol Sci. (2023) 24:12483. doi: 10.3390/ijms241512483, PMID: 37569857 PMC10419971

[22] Dayer J-M Oliviero F Punzi L. A brief history of IL-1 and IL-1 Ra in rheumatology. Front Pharmacol. (2017) 8:293. doi: 10.3389/fphar.2017.00293, PMID: 28588495 PMC5440542

[23] McGeachy MJ Cua DJ Gaffen SL. The IL-17 family of cytokines in health and disease. Immunity. (2019) 50:892–906. doi: 10.1016/j.immuni.2019.03.021, PMID: 30995505 PMC6474359

[24] Kleinschek MA Owyang AM Joyce-Shaikh B Langrish CL Chen Y Gorman DM. IL-25 regulates Th17 function in autoimmune inflammation. J Exp Med. (2007) 204:161–70. doi: 10.1084/jem.20061738, PMID: 17200411 PMC2118427

[25] von Moltke J Ji M Liang HE Locksley RM. Tuft-cell-derived IL-25 regulates an intestinal ILC2-epithelial response circuit. Nature. (2016) 529:221–5. doi: 10.1038/nature16161, PMID: 26675736 PMC4830391

[26] Vilstrup H Amodio P Bajaj J Cordoba J Ferenci P Mullen KD. Hepatic encephalopathy in chronic liver disease: 2014 Practice Guideline by the American Association for the Study of Liver Diseases and the European Association for the Study of the Liver. Hepatology. (2014) 60:715–35. doi: 10.1002/hep.27210, PMID: 25042402

[27] Angeli P Gines P Wong F Bernardi M Boyer TD Gerbes A. Diagnosis and management of acute kidney injury in patients with cirrhosis: revised consensus recommendations of the International Club of Ascites. Gut. (2015) 64:531–7. doi: 10.1136/gutjnl-2014-308874, PMID: 25631669

[28] Jalan R Saliba F Pavesi M Amoros A Moreau R Ginès P. Development and validation of a prognostic score to predict mortality in patients with acute-on-chronic liver failure. J hepatology. (2014) 61:1038–47. doi: 10.1016/j.jhep.2014.06.012, PMID: 24950482

[29] Miossec P Korn T Kuchroo VK. Interleukin-17 and type 17 helper T cells. New Engl J Med. (2009) 361:888–98. doi: 10.1056/NEJMra0707449, PMID: 19710487

[30] Beringer A Miossec P. IL-17 and IL-17-producing cells and liver diseases, with focus on autoimmune liver diseases. Autoimmun Rev. (2018) 17:1176–85. doi: 10.1016/j.autrev.2018.06.008, PMID: 30321671

[31] Cerboni S Gehrmann U Preite S Mitra S. Cytokine-regulated Th17 plasticity in human health and diseases. Immunology. (2021) 163:3–18. doi: 10.1111/imm.13280, PMID: 33064842 PMC8044333

[32] Schnell A Littman DR Kuchroo VK. TH17 cell heterogeneity and its role in tissue inflammation. Nat Immunol. (2023) 24:19–29. doi: 10.1038/s41590-022-01387-9, PMID: 36596896 PMC10795475

[33] Chung S-H Ye X-Q Iwakura Y. Interleukin-17 family members in health and disease. Int Immunol. (2021) 33:723–9. doi: 10.1093/intimm/dxab075, PMID: 34611705 PMC8633656

[34] Kuestner RE Taft DW Haran A Brandt CS Brender T Lum K. Identification of the IL-17 receptor related molecule IL-17RC as the receptor for IL-17F. J Immunol. (2007) 179:5462–73. doi: 10.4049/jimmunol.179.8.5462, PMID: 17911633 PMC2849293

[35] Toy D Kugler D Wolfson M Bos TV Gurgel J Derry J. Cutting edge: interleukin 17 signals through a heteromeric receptor complex. J Immunol. (2006) 177:36–9. doi: 10.4049/jimmunol.177.1.36, PMID: 16785495

[36] Matsuda KM Kotani H Hisamoto T Kuzumi A Fukasawa T Yoshizaki-Ogawa A. Dual blockade of interleukin-17A and interleukin-17F as a therapeutic strategy for liver fibrosis: Investigating the potential effect and mechanism of brodalumab. Cytokine. (2024) 178:156587. doi: 10.1016/j.cyto.2024.156587, PMID: 38531177

[37] Huang L Liu J Bie C Liu H Ji Y Chen D. Advances in cell death-related signaling pathways in acute-on-chronic liver failure. Clinics Res Hepatol Gastroenterology. (2022) 46:101783. doi: 10.1016/j.clinre.2021.101783, PMID: 34339873

[38] Zhu H Sun B Shen Q. TNF-α induces apoptosis of human nucleus pulposus cells via activating the TRIM14/NF-κB signalling pathway. Artif cells nanomedicine Biotechnol. (2019) 47:3004–12. doi: 10.1080/21691401.2019.1643733, PMID: 31322007

[39] Yang XO Chang SH Park H Nurieva R Shah B Acero L. Regulation of inflammatory responses by IL-17F. J Exp Med. (2008) 205:1063–75. doi: 10.1084/jem.20071978, PMID: 18411338 PMC2373839

[40] Gomez-Rodriguez J Sahu N Handon R Davidson TS Anderson SM Kirby MR. Differential expression of IL-17A and IL-17F is coupled to TCR signaling via Itk-mediated regulation of NFATc1. Immunity. (2009) 31:587. doi: 10.1016/j.immuni.2009.07.009, PMID: 19818650 PMC2767186

[41] Wu M-S Wang C-H Tseng F-C Yang H-J Lo Y-C Kuo Y-P. Interleukin-17F expression is elevated in hepatitis C patients with fibrosis and hepatocellular carcinoma. Infect Agents Cancer. (2017) 12:1–6. doi: 10.1186/s13027-017-0152-7, PMID: 28770001 PMC5530479

[42] Andres-Hernando A Dursun B Altmann C Ahuja N He Z Bhargava R. Cytokine production increases and cytokine clearance decreases in mice with bilateral nephrectomy. Nephrol Dialysis transplantation. (2012) 27:4339–47. doi: 10.1093/ndt/gfs256, PMID: 22778179 PMC3520082

