# Supplementary material for: Serum IL-17F as a biomarker of infection-independent cirrhosis progression
Source: Front Immunol. 2025 Oct 22;16:1671288. doi: 10.3389/fimmu.2025.1671288 (PMC12585995; doi:10.3389/fimmu.2025.1671288)

Supplementary Table 1: *Univariable regression evaluating the association between circulating interleukin levels and 12-week mortality.*

**Variable**	**Odds Ratio (OR)**	**95% Confidence Interval**	**p-value**
IL-23 (log)	0.78	0.39-1.19	0.34
IL-17A (log)	1.19	0.79-1.66	0.341
IL-17F (log)	0.96	0.64-1.36	0.846
IL-17E (log)	0.94	0.55-1.38	0.773
IL-1RA (log)	1.09	0.74-1.62	0.669
IL-1β (log)	0.82	0.40-1.28	0.474

Supp. Figure 1. Univariable Ordninal regression

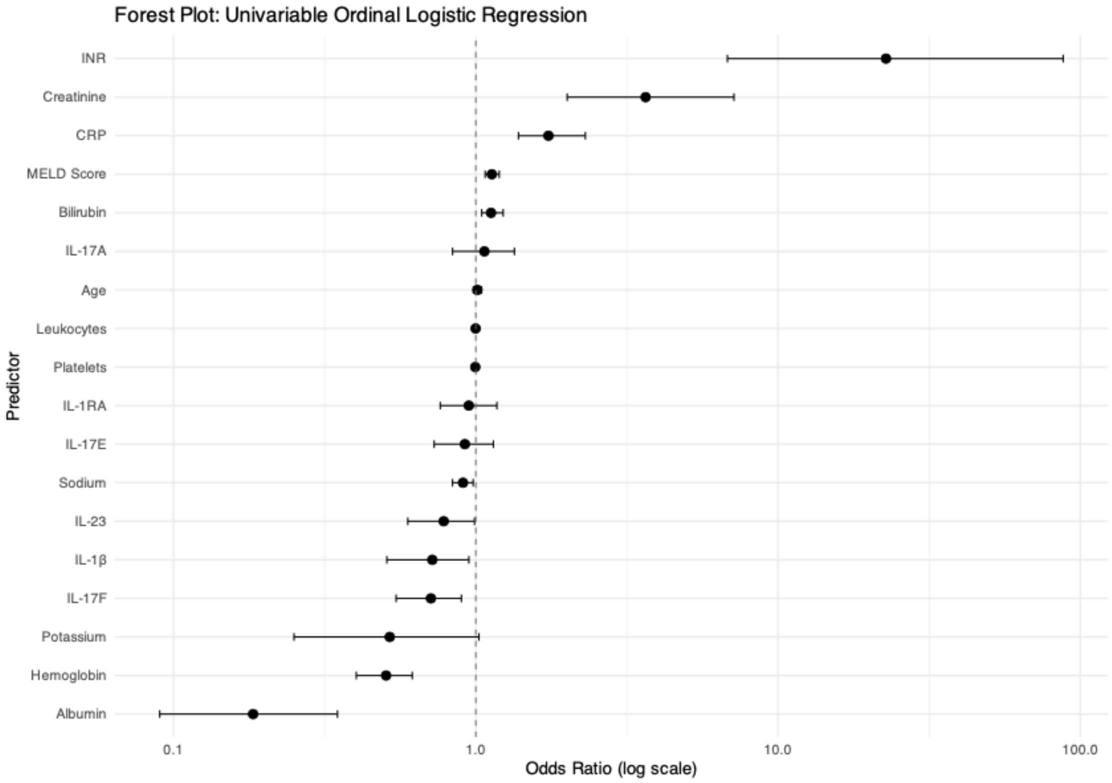

Supp. Figure 2. Spearman correlation matrix of baseline cytokines and standard clinical laboratory markers

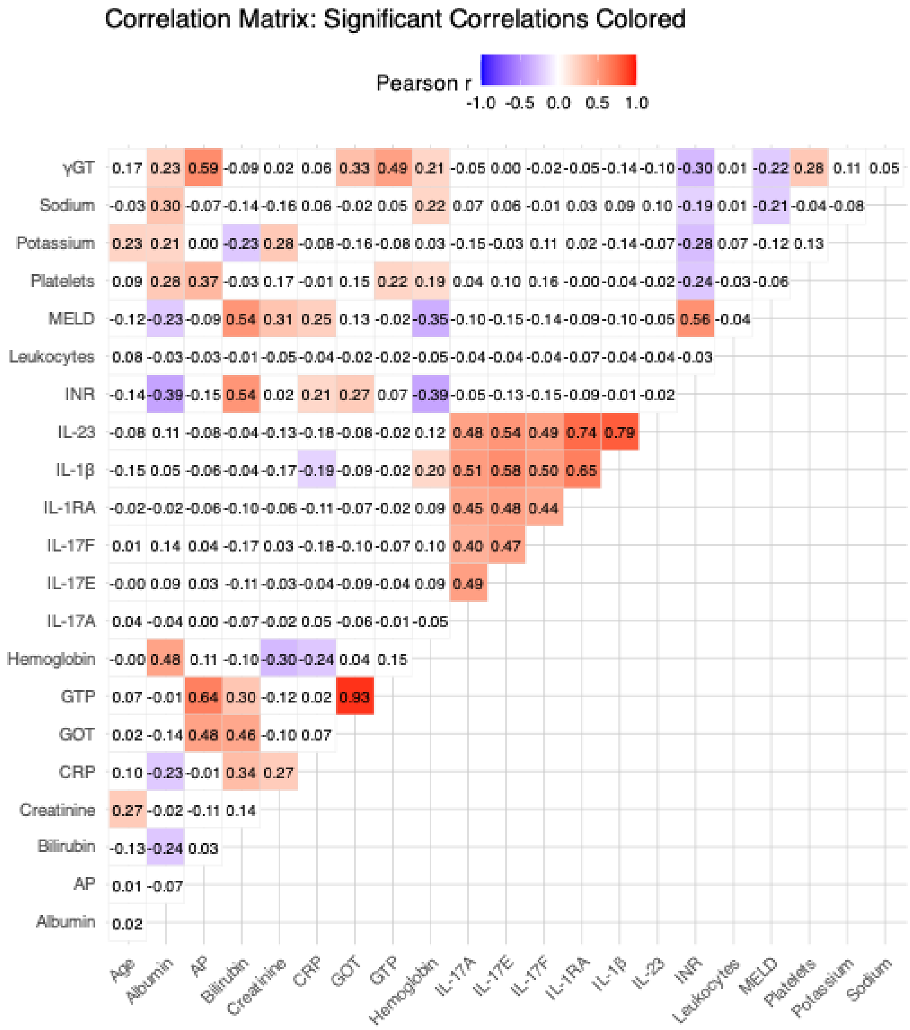

Supp. Figure 3. Spearman correlation between the cytokine levels

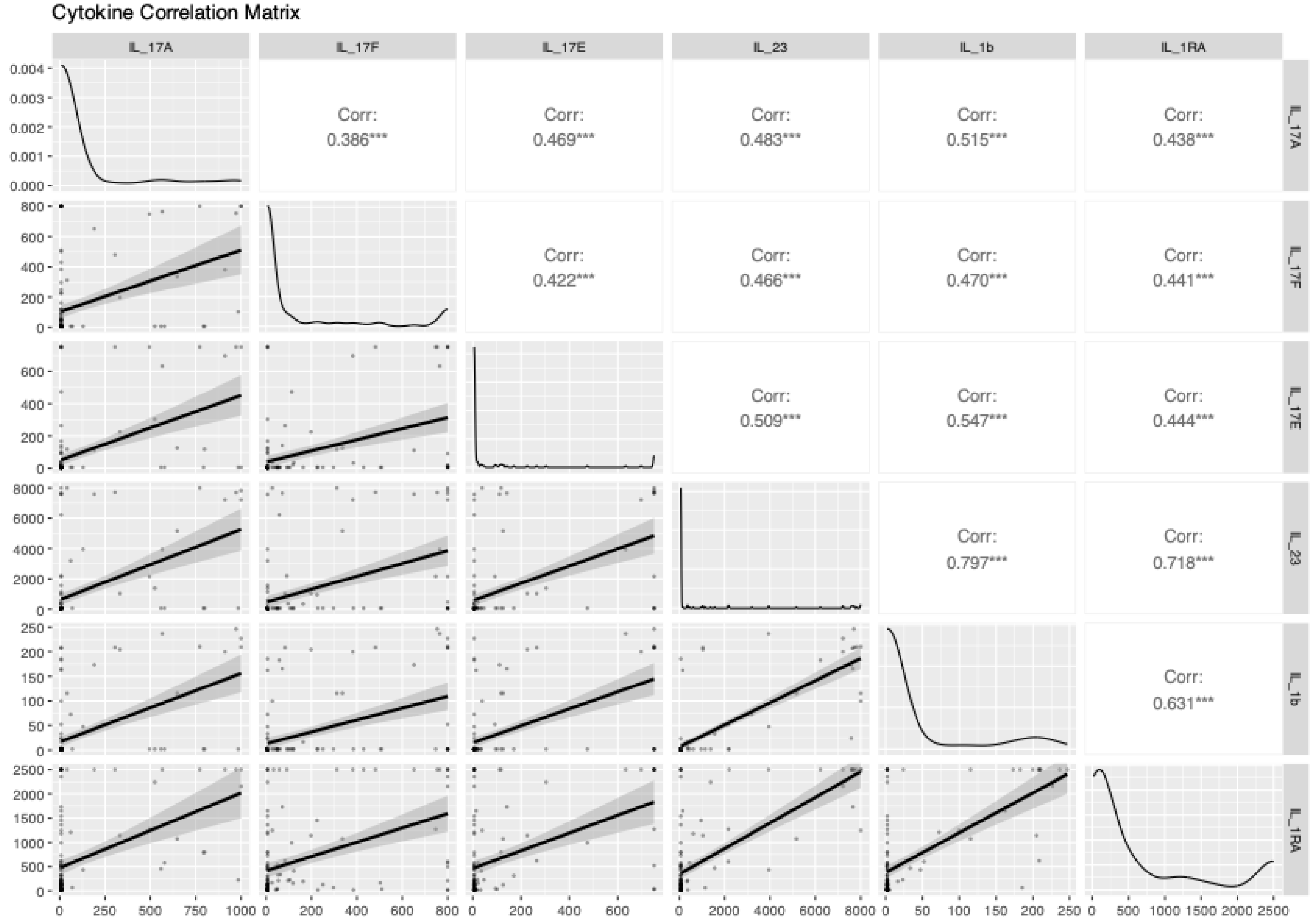

Supplement: Supplementary Table 1 — Univariable regression evaluating the association between circulating interleukin levels and 12-week mortality. [file Image1.pdf]
